# Self-efficacy assessment hinders improvement on a deliberate cricket bowling practice task

**DOI:** 10.3389/fpsyg.2023.1214767

**Published:** 2023-09-19

**Authors:** Dhruv Raman, Bittu Rajaraman

**Affiliations:** ^1^Department of Psychology, Ashoka University, Sonepat, Haryana, India; ^2^Wheelock College of Education, Boston University, Boston, MA, United States

**Keywords:** self-efficacy, assessment, cricket, motor learning, deliberate practice

## Abstract

**Introduction:**

Previous research indicates that external focused attention is linked to superior performance on motor tasks. This study examined how attention directed toward one’s self-efficacy affected performance in a cricket bowling task.

**Methods:**

In the pre-test phase, participants attempted to bowl in a designated “good length” zone across 12 trials. Following this, participants were randomly assigned to either an experimental group, where they rated their own general and task-specific self-efficacy, or a control group, where they rated someone else’s ability. They each then bowled 12 more trials. Their performance was measured based on the number of trials that were bowled within the standard “good length” zone.

**Results:**

Paired t-tests showed that while the performance of the control group improved significantly from pre-test to post-test, *t* = 2.613, *p* = 0.008; the experimental group did not show a significant improvement, *t* = 1.156, *p* = 0.131.

**Discussion:**

Results indicate that asking people to rate their self-efficacy level may reduce their improvement on a deliberate practice task. Implications for sport performance and researchers are discussed.

## Introduction

Does reflection on one’s own self-efficacy negatively impact improvement on a motor task? Bandura (2006) argues that administering a self-efficacy assessment is unlikely to change behavior. However, researchers who have examined sledging show that external activation of cognitive processes in athletes can influence their performance through attentional distraction and initiation of negative emotion (Davis et al., 2018). In the absence of social actors like opponents or coaches, this study examined whether simply asking athletes to rate their self-efficacy hindered their improvement on a deliberate practice motor task. The research has implications for how cognitive processes might relate to improvement on motor tasks.

### Self-efficacy, deliberate practice, and motor learning

Self-efficacy refers to someone’s belief in their ability to successfully execute a task. It differs marginally from confidence in that one could be confident about not doing well on a task (Bandura, 1997, 2006; Short and Ross-Stewart, 2008). Self-efficacy beliefs can be affected by past performance, vicarious experiences, motivational experiences, physiological states, and verbal persuasion, and can in turn impact future actions, choices, effort and persistence (Bandura, 1997). The positive relationship between self-efficacy and motor task performance has been well-documented (Moritz et al., 2000; Anstiss et al., 2020). Specifically, having higher self-efficacy is usually a good indication of how someone might perform on a motor task.

Previous studies have looked at self-efficacy in the context of motor learning. In a study on transfer of learning, Stevens et al. (2012) found that self-efficacy assessment prior to a post-test task was the strongest predictor of performance on that task. Stevens et al. argue that self-efficacy acts as a mediator for learning on a task, i.e., a participant’s initial performance is linked to their subsequent self-efficacy report, which in turn is linked to their subsequent performance. This study aimed to examine if this link could limit improvement on a task which otherwise prompts self-improvement.

Deliberate practice tasks are designed to support improvement in performance across various domains such as sports, music, and typing (Ericsson, 2008; Coughlan et al., 2014). In deliberate practice tasks, performers isolate one specific component of a task and use repetition to improve on that task (Ericsson, 2008). Researchers find that improvements on such tasks are stronger when players are allowed to generate their own movements to reach a sporting goal, compared to when components of the movement are guided by feedback from experts (Soderstrom and Bjork, 2015).

While deliberate practice tasks are geared to help individuals improve, activation of certain cognitive processes may interfere with learning. In a study examining limits to self-guided improvement on a motor task, Chiviacowsky et al. (2012) manipulated the criterion of what was considered *good* performance. The participants who had a harder criterion for good performance, showed a drop in self-efficacy levels and poorer performance on a post-test and 24-h retention task. Chiviacowsky et al. concluded that participants’ experience of competence, noted through self-efficacy assessments, was an important part of learning outcomes. What happens when individuals are forced to reflect on their competence while learning?

One mechanism through which reflection self-efficacy assessment may hinder improvement is internal attentional focus. Attentional focus is one of the primary factors that distinguishes better performances on motor tasks from worse ones (Memmert, 2009; Porter et al., 2010; Abdollahipour et al., 2016; Wulf et al., 2018). Multiple meta-analyses have revealed that an external focus away from one’s body is linked to superior motor performance and motor learning (Wulf, 2013; Chua et al., 2021). The locus of attention also affects outcomes, with a focus on more distal loci to the body associated with better performances than focusing upon more proximal loci, as assessed in a variety of sports involving manipulating the body in space (Bell and Hardy, 2009). Self-efficacy assessments ask athletes to reflect on their skills and abilities and may prime athletes to focus on their internal bodily movements. We hypothesize that such reflection will, in turn, hinder improvement on a motor task.

### The present study

In order to explore the specific effect of realistic feedback on self-efficacy and performance, this study examined Indian cricketers, a sport with pauses built in after each period of play during which attentional focus might shift toward various inward factors. Much of the scientific literature on cricket has examined the physiological characteristics of differently skilled players (Edwards and Beaton, 1996; Barrett et al., 2017), the biomechanics of batting (Taliep et al., 2007) and bowling (Renshaw and Davids, 2006; Callaghan et al., 2018), the effects of pre-performance routines (Cotterill, 2011), techniques (Wallis et al., 2002), and training methods (Ford et al., 2010; Low et al., 2013). Most psychology of cricket literature has looked at differences between players in anticipation (Ford et al., 2010), head and eye movements while batting (Sarpeshkar et al., 2017), judgment of ball length and direction (Müller et al., 2006), ability to discriminate among various delivery types (Renshaw and Fairweather, 2000), and stress and coping strategies (Thelwell et al., 2007). While cricket is among the most popular sports in the world, it remains underexplored in the psychology literature.

This study proposes to examine performance before and after a manipulation midway through a standardized bowling accuracy task. Self-related thoughts are elicited by administering a self-efficacy questionnaire where players reflect on their performance and are given knowledge of their results. Players in a control group are asked to reflect on the performance of another bowler. All participants are not given any technical instructions and are allowed to improve through self-generated movements. This study examines how prompting athletes to think about their skills and abilities, through rating one’s self-efficacy, in the middle of a deliberate practice task influences performance. We hypothesize that priming self-related thoughts in the middle of a deliberate practice task is likely to hinder improvement on the task.

## Methods

### Participants

Participants were recruited from a Private University and two cricket academies from the National Capital Region in India. All athletes who were able to bowl and present on practice on the days of the experiments were offered the ability to participate. Participants were between 16 and 24 years of age (*M* = 18.48, *SD* = 1.95) and included 5 female and 37 male cricketers who had training experience ranging from less than 1 year to more than 5 years. During recruitment, participants were told that the study was about learning a specific bowling skill. Informed consent was taken from the participants, and in the case of minors, from their coaches. Some participants were told that they will be rewarded with food coupons (*N* = 38), while others were promised extra credit (*N* = 4). The research design was approved by the Institutional Review Board of Ashoka University.

### Set-up

**Figure 1:**
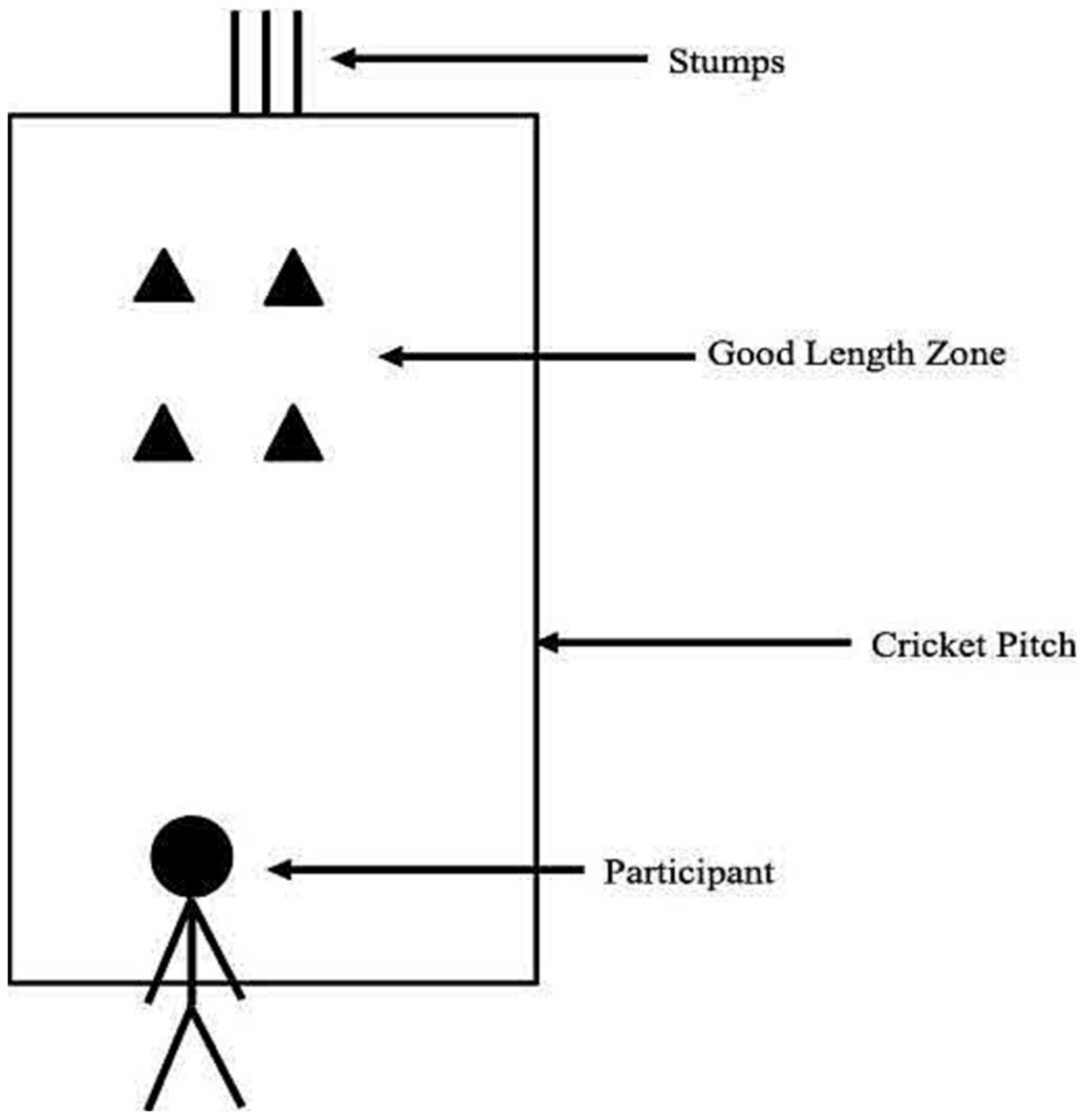
Experimental set-up. The bowlers attempted to pitch the ball within the *“Good Length Zone”* area demarcated by the cones, indicated by the triangles, in a standard sized cricket pitch which was replicated in the experiment. The *“Good Length Zone”* area was between four and six meters from the stumps.

An illustration of the set-up is shown in Figure 1. The experiment was conducted in standard cricket nets used for training. The surface of the pitch was either concrete or soil — based on availability at the cricket academy. While a change in surface may have impacted what the ball did after pitching, this did not impact their performance on the task — which was only concerned with where the ball pitches. Three stumps were placed on the closed end of the net and one stump on the bowling end. The distance between the stumps, batting crease and bowling crease was marked according to standard cricketing dimensions. Additionally, saucer cones were placed at four and six meters away from the stumps at the batting end. This distance is typically used to indicate a “good length zone” — an area where trained bowlers attempt to bowl to create difficulty for batsman. This zone was two meters long and half a meter wide. It started from the right most stump (leg stump for a right hander) and ended slightly outside the left-most stump. This was to ensure that bowling accuracy is measured on both parameters: line and length which typically determine the quality of a ball. The outside vertices of this zone was marked using four saucer cones. An experimenter stood on the side of the cricket net to check for no-balls (see “Invalid Trials”) and to determine where the ball pitched. Video cameras were placed behind the stumps to corroborate the experimenter’s observation.

### Measures

#### Task performance

A simple “hit or miss” paradigm was used and trials were deemed successful if the ball pitched inside the target zone and unsuccessful otherwise.

#### Invalid trials

These included (a) front foot no balls: trials that were bowled from in front of the bowling crease (no part of the leading foot of the player is behind the line); (b) throwing no balls: trials where participants bowled with a bent arm (at an angle greater than 15 degrees). These trials were not counted during pre-test or post-test phase. This was checked by an experimenter.

#### Training experience

Training experience was measured using three questions. These asked about years of formal cricket training an individual had received, how many hours of such training they received in a week and how many hours they practiced bowling in a week. Formal cricket training was defined as any training received from a coach at school, university or a cricket academy. The last two questions were answered in relation to the last three months to gage present involvement in cricket.

#### Self-efficacy

Self-efficacy was measured using Bandura’s 100 point scale using two items. This scale has been used for self-efficacy measurement in a sporting context (see Treasure et al., 1996). The scale indicates confidence levels from “not at all confident” (0) to “moderately confident” (50) to “very confident” (100). The first questions asked about participant’s confidence about their bowling. The second question asked how confident the participants were of bowling 50% of the deliveries in the target zone. This item was adapted from Ong et al. (2015) who used a similar measure as task self-efficacy indicator for dart throwing. The two questions gave an indication of participant’s general and task self-efficacy, respectively. Only two items were used to understand whether even a brief period of attention toward one’s self-efficacy may impact performance, as well as to make the self-efficacy intervention seem less obtrusive.

#### Control group questionnaire

Participants (*N* = 21) were asked a set of questions – they were asked first to write the name of their favorite bowler. Then, they too were given a 100-point scale and asked to rate their favorite bowler’s ability on this scale. This second version of the control group questionnaire was to ensure that the question and responses of the control group are similar to the experimental group in all but one aspect — they do not prompt their attention to be directed inwards to the self.

### Procedure

The experiment had a mixed design and was divided into three phases. Overall, the participants had to bowl 24 times. Pilot studies that we conducted showed that this was an ideal number of deliveries to measure improvement while preventing fatigue — especially for fast bowlers (see Maunder et al., 2017). After the first 12 balls (Phase 1), participants were given a five minute break and they were administered a questionnaire (Phase 2). Half the participants (*N* = 21) were administered the control group questionnaire, while the other half was administered the experimental group questionnaire. Finally, after they bowled 12 more times, they were administered another questionnaire, were thanked, rewarded and debriefed (Phase 3). The entire task was conducted on the same day and took between 20 and 30 min for each participant.

#### Phase 1

This phase constituted of warm up and 12 bowling trials. First, participants were asked to read and sign the informed consent. Then, they were told to stretch and warm up. As part of the warm up, they were asked to bowl three times without the cones in place. During these trials, the experimenter gave them feedback about invalid trials (if any) and they were required to correct this before they began. No participant required more than three warm up deliveries to correct invalid trials. After the warm up, participants were given instructions in either English or Hindi depending on their comfort. Following the instructions, the participants proceeded to bowl two blocks of six trials each. They could take a 30 s break after the first six balls.

#### Phase 2

After the first 12 balls, participants were told how many trials were successful. This was conveyed verbally by the experimenter in a neutral tone. For example, “Four out of 12 trials pitched inside the zone.” While the results were visible in plain sight to the participants, the feedback was made explicit to ensure that knowledge of results is uniform across participants. This was important because their results may have affected their self-efficacy ratings. Then, they were given a break of 5 min. In this break they were allowed to drink water as lack of hydration has been shown to influence bowling accuracy (Devlin et al., 2001). However, they were not allowed to consume any other substances. After 5 min, both groups were administered “Questionnaire A” which asked about the duration and frequency of cricket training. Then, the experimental manipulation was introduced. While experimental group participants (*N* = 21) were administered “Questionnaire B,” control group (*N* = 21) participants were administered “Questionnaire C.” The participants were assigned to these groups through random number generation using the software R.

#### Phase 3

Following phase 2, the participants bowled one warm up delivery without the cones. This was because pilot studies had shown that participants required at least one warm up delivery to start bowling accurately again. Then, they were given the same instructions to bowl for the next set of trials. Following this, the participants bowled 12 times with an optional 30 s break after six balls. The criterion to indicate the success of a trial was the same as Phase 1. At the end of these trials, all participants were told the number of successful trials in the last 12 balls. Then, participants from both groups were administered “Questionnaire B.” This was to get an after task self-efficacy measure from both groups and also to see if participants in the experimental group changed their self-efficacy scores. Finally, the participants were thanked, rewarded and debriefed regarding the study. In the debrief they were told about the aims of the study, the primary research question and the hypothesis.

## Results

### Main analyses

The primary hypothesis was tested using a paired t-test for control and experimental groups, based on a 2 × 2 design: (Test Time: Pre-test vs. Post-test × Questionnaire Administered: Control vs. Experimental) design. The performance variable (DV) was the number of times the participants “hit” the good length zone in the 12 pre-test trials versus the 12 post-test trials. Table 1 shows the descriptive statistics for the performance of bowlers, separately for the control and experimental groups.

**Table 1:** Descriptive statistics for successful trials separated by group.

Group	Control group			Experimental group		
	Pre-test	Post-test	Overall	Pre-test	Post-test	Overall
Participants	21	21	21	21	21	21
Trials	6	6	12	6	6	12
Mean	3.05	4.14	7.19	3.14	3.61	6.76
Std. Deviation	1.50	1.53	2.34	1.90	1.82	3.22
Minimum	1	1	3	0	1	2
Maximum	7	7	11	7	8	14

Trials indicates the total number of deliveries bowled by each participant. Mean score, standard deviation score, minimum and maximum is for the number of successful trials pooled across participants for each group.

**Figure 2:**
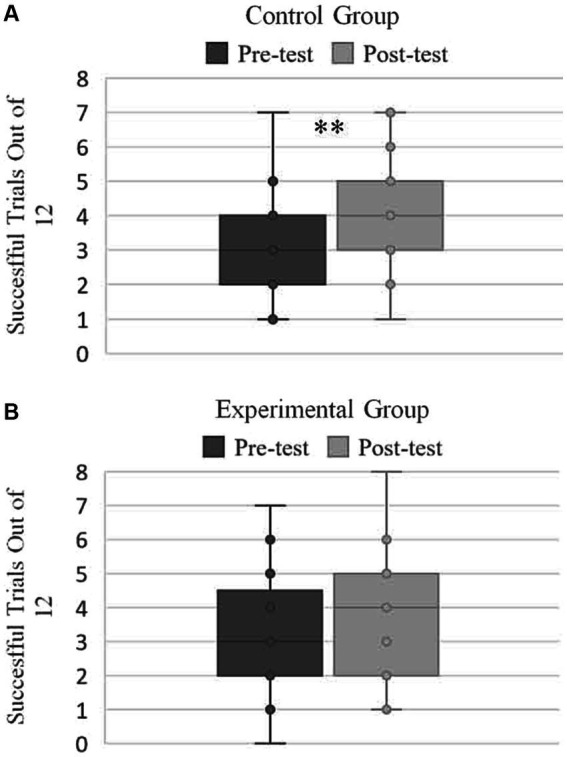
Improvement in performance. Pre-test indicates the number of successful trials in the first set of 12 trials, whereas, Post-test indicates the number of successful trials in the second set of 12 trials. **(A)** Mean comparisons for pre-test and post-test in the Control Group. **(B)** Mean comparisons for pre-test vs. post-test in Experimental Group.

The Shapiro–Wilk normality test showed that pre-test scores for the control group (*W* = 0.92, *p* = 0.10) and experimental group (*W* = 0.95, *p* = 0.29) were normally distributed; as were the post-test scores for the control group (W = 0.95, *p* = 0.34) and experimental group (W = 0.95, *p* = 0.34). Post-test bowling scores (*M* = 4.14, *SD* = 1.53) were significantly higher than the pre-test bowling scores (*M* = 3.05, *SD* = 1.50) for the control group, *t*(20) = 2.61, *p* = 0.008. However, post-test bowling scores (*M* = 3.619, *SD* = 1.830) for the experimental group were not significantly greater than the pre-test bowling scores (*M* = 3.14, *SD* = 1.91), *t* (20) = 1.16, *p* = 0.131 (Figure 2). This supported the primary hypothesis which stated that improvement would be less for the experimental group in comparison to the control group.

A Mixed ANOVA showed that there was a significant main effect of test time, *F* (1, 40) = 7.15, *p* < 0.05. This means that there was an overall significant improvement for all bowlers from pre-test to post-test confirming the known effects of practice on producing improvement. However, the interaction of test time and questionnaire administered was not significant, *F* (1, 40) = 1.11, *p* = 0.298. This indicates that the improvement did not simplistically interact with the intervention.

We checked that there were no initial differences between the two groups, who were randomly drawn from the test population. An independent samples t-test indicated that there was no significant difference in the pre-test scores for experimental vs. control group, *t*(40) = −0.180, *p* = 0.858. Similarly, there was no significant difference in post-test scores for experimental vs. control group, *t* (40) = 1.01, *p* = 0.160. Additionally, there was no significant difference between overall scores for the experimental group (*M* = 7.19, *SD* = 2.34) and the control group (*M* = 6.76, *SD* = 3.22), *t* (40) = 0.493, *p* = 0.625. This meant that either before or after the intervention, there were no differences in average performance scores of individuals across the two groups. Further, absolute change in scores were not significantly lower for the experimental group (*M* = 0.476, *SD* = 1.89) compared to the control group (*M* = 1.10, *SD* = 1.92), *t* (40) = 1.05, *p* = 0.149. This indicated that when change in scores is used as the dependent variable, the difference between the two groups was not significant.

### Self-efficacy

There were two measures of self-efficacy. One measured general self-efficacy as a bowler, whereas the other measured task-specific self-efficacy. For the experimental group, these measures were taken twice: once after the pre-test and again after the post-test. However, for the control group these were only taken after the post-test. Separate analyses were conducted for the two groups since there was a difference in when self-efficacy scores were measured.

### Self-efficacy and change in scores

Interestingly, for the experimental group, the absolute change in bowling scores was negatively correlated to both the general self-efficacy scores (*r*(19) = −0.51, *p* = 0.017) and to task-specific self-efficacy (*r*(19) = −0.45, *p* = 0.043) measured immediately after pre-test bowling. This meant that higher self-efficacy was linked to a lower likelihood of improvement on the post-test. The correlation between the absolute change in bowling scores with general and task specific efficacy measured after post-test bowling, in both the experimental and control groups, was negative but not significant. When participant’s performance scores were pooled across the experimental and control groups, the overall showed a moderate positive correlation with both general (*r*(40) = 0.41, *p* = 0.007) and task self-efficacy (*r*(40) = 0.43, *p* = 0.005). Table 2 shows the correlations for training experience, self-efficacy, and performance pooled across the control and experimental groups.

**Table 2:** Correlational matrix for experimental and control group scores combined.

	Years of training	Weekly hours	Weekly bowling hours	General self-efficacy	Task self-efficacy	Overall score	Absolute change	Pre-test
Hours in a week	0.244	—						
	0.119	—						
Bowling in a week	0.217	0.742	—					
	0.168	< 0.001	—					
General self-efficacy	0.494	0.598	0.647	—				
	< 0.001	< 0.001	< 0.001	—				
Task self-efficacy	0.305	0.549	0.595	0.668	—			
	0.050	< 0.001	< 0.001	< 0.001	—			
Overall score	0.214	0.372	0.390	0.413	0.425	—		
	0.174	0.015	0.011	0.007	0.005	—		
Absolute change	−0.269	−0.294	−0.162	−0.262	−0.228	−0.006	—	
	0.085	0.058	0.306	0.094	0.147	0.972	—	
Pre-test	0.327	0.472	0.412	0.488	0.478	0.827	−0.567	—
	0.034	0.002	0.007	0.001	0.001	< 0.001	< 0.001	—
Post-test	0.025	0.142	0.231	0.194	0.223	0.825	0.561	0.363
	0.877	0.371	0.141	0.219	0.155	< 0.001	< 0.001	0.018

The first row next to each variable refers to Pearson’s *r*-correlation value and the second row refers to the *p*-value. Weekly hours refer to hours of cricket training, generally, and Weekly bowling hours refer to hours of bowling training in a week. The general self-efficacy and task self-efficacy scores are pooled for the measures that were taken after the post-test for both groups. The self-efficacy measures which were taken only for the experimental group prior to the post-test were part of the manipulation and are not included. Absolute change refers to the absolute difference in scores between post-test and pre-test.

### Training experience and test scores

There were three different measures of training experience: number of years of cricket training one has received; number of hours one receives such training in a week (for the last three months); and the number of hours one practices bowling in a week (for the last three months). Based on participant ratings, the scores for all items about training experience were normalized to a number from 1 to 5. Correlation analyses were run to see how training experience interacts with other variables.

A weak positive correlation emerged between various measures of training experience and performance on the task. Years of training an individual received (*Mean normalized score* = 2.79, *SD* = 1.14) was positively correlated with their pre-test scores *r*(40) = 0.33, *p* = 0.034, when participants were pooled across the experimental and control groups. Similarly, pre-test scores were also positively correlated with hours of cricket training in a week (*Mean normalized score* = 3.36, *SD* = 1.51), *r*(40) = 0.47, *p* = 0.002; and number of hours one practiced bowling in a week (*Mean normalized score* = 2.69; *SD* = 1.41), *r*(40) = 0.41, *p* = 0.007. However, this correlation was not significant for post-test scores, either when disaggregated for control and experimental groups, or when pooled together.

### Gender

There were only five female participants compared to 37 male participants. Due the large variation in sample sizes, it is difficult to draw any meaningful conclusions from this aspect of the data. Data for pre-test and post-test scores pooled together, and pooled across experimental and control groups and disaggregated by gender showed that the mean self-efficacy scores on the task were lower for women (*M =* 51; *SD* = 23.02) than for men (*M* = 67.20; *SD* = 26.74). This is despite the fact that women participants had higher mean overall bowling scores (*M* = 9, *SD* = 2.12) than male participants (*M* = 6.70, *SD* = 2.778). Similarly, the absolute change in scores was higher for women (*M* = 2.2, *SD* = 1.64) than men (*M* = 0.595, *SD* = 1.88). However, an ANOVA indicated that neither the difference in bowling scores (*F*(1, 40) = 3.144, *p* = 0.084) nor self-efficacy (*F*(1, 40) = 1.66, *p* = 0.205) between men and women was statistically significant. Pre-test scores pooled across experimental and control groups did not lend themselves to statistical tests requiring normal distributions, since they did not show normality when disaggregated by gender and checked with a Shapiro–Wilk test.

## Discussion

This study showed that cricket bowlers who were asked to rate their self-efficacy scores in the middle of a deliberate practice task, showed no significant improvement in performance, compared to a control group which showed a significant improvement, across two sets of trials in a bowling accuracy task. These results support the hypothesis that a self-efficacy assessment might interfere with learning on a motor task. However, a comparison using the difference of means between groups was not significant. These findings require further examination with more participants to make substantial claims.

These results suggest that reflecting on one’s competence may hinder immediate improvement during a task. The manipulation included two items. The first item asked about an individual’s self-efficacy levels as a bowler, while the second rating asked how confident they were of bowling 50% of the balls in the target zone. Both these questions require athletes to make estimates about their own ability and performance. In the absence of such estimates, the athletes were able to improve their performance on the task.

These findings show a similar pattern to Stevens et al.’s (2012) study where participants’ self-efficacy assessments immediately before a trial were the strongest predictor of their performance, explaining 55% of the variance in performance. The findings of the present study indicate that the strong relationship between self-efficacy assessments and performance may make the assessments a hindrance to improvement. These findings diverge from Bandura’s (2006) claims that self-efficacy assessments themselves can influence behavior.

The lack of improvement in the experimental group may be explained through two potential mechanisms. One mechanism is that performance expectancies might be strengthened through a self-efficacy assessment. Athletes may rate their self-efficacy in line with how they performed on the pre-test and create performance expectancies in line with their pre-test performance. These limited performance expectancies may, in turn, limit improvement. Without the administration of a self-efficacy questionnaire—as was the case with the control group—these performance expectancies are not similarly strengthened.

A second potential mechanism to explain these results is that self-efficacy assessments prompted internal attentional focus. When asked to reflect on their self-efficacy, athletes may be *prompted* to think about their skills and abilities and in turn focus on their body movements while executing the task. Internal attentional focus is linked to poorer performance on motor tasks for both short-term and long-term motor learning (Wulf, 2013; Chua et al., 2021).

It is also possible that these two mechanisms have an additive impact, i.e., self-efficacy assessments limit performance expectancies and initiate an internal focus of attention during the task. Wulf et al. (2018) reported a positive additive effect of enhanced performance expectancies and external attentional focus on performance. Future researchers can examine if similar additive effects can also inhibit performance through trends shown in the present study.

An alternate explanation for the results is that the control group improved more because of the questions administered to them. Simply thinking about their favorite bowler could have enhanced their performance on the post-test. Previous research has looked at related occurrences. For instance, in a study with golfers, it was found that participants who were told that their equipment belonged to a pro-golfer previously, performed better than a control group (Lee, 2012). However, a key aspect of the participants’ performance — the equipment — was linked to the pro-golfer. Such a link was missing in the present experiment.

Two other patterns of results are worth discussing. First, self-efficacy measures were shown to be highly positively correlated to overall scores in both groups. This aligns with several studies which continue to show a strong relationship between self-efficacy and performance (Anstiss et al., 2020). Second, in a small sample of five female participants, the results indicated a trend that average self-efficacy estimates might be lower than the sample of men, despite higher average scores. This trend of lower self-efficacy reporting is in line with previous research (see Feltz, 1988; Wang et al., 2020). Future studies can examine differences in self-efficacy estimates between athletes who have similar skill levels but belong to different genders.

## Implications

The findings of this study have implications for how cognitive processes might influence improvement on a motor task. The primary implication is that mid-performance self-evaluation might be detrimental to motor improvement. An athlete may be tempted to think about internal psychological factors such as their self-efficacy, or may be prompted to do so by a coach or an opponent. These may set limiting performance expectations, prompt internal attentional focus, or both. Either way, the results of this experiment indicate that activating a cognitive process related to self-evaluation might be detrimental to improvement on a motor task. Future studies can use longitudinal designs to examine whether self-reflection similar effects are seen on long-term improvement as well.

This study also has implications for researchers studying cognitive processes in real-world settings. As discussed earlier, self-efficacy is measured using questions like those used in this experiment. Results showed that simply using a two-item scale, such questions may hinder an athlete’s improvement on the consequent task. Thus, researchers may want to account for the effects of these questionnaires on performance. This is particularly important when athletes are asked to answer these questions in the middle of the task, i.e., after receiving feedback.

### Limitations and future directions

While the findings of this experiment add to previous research, it has some limitations. First, the intervention only included two questions as a measure of self-efficacy. Thus, this may have prevented us from gaining a holistic understanding of the participant’s self-efficacy. Further, the only difference between the two groups was in the form of these two questions administered to them. A minimal number of questions were used to replicate minor prompts that may cause such reflections in real life contexts and to reduce the obviousness of the intervention. Self-reflective thoughts may be fleeting in sporting contexts where attention is captured by a variety of stimuli. The use of two items indicates that even a slightly prolonged attention to these thoughts may be detrimental to one’s performance. It is likely that if there were more questions that made participants reflect in greater detail, the results would have been different. Future studies that measure performance and self-efficacy in a more continuous way, or along a wider spectrum, may lead to a more nuanced and comprehensive understanding of the relationship between bowling accuracy and self-efficacy.

The second limitation is regarding the results for the between group comparisons. The hypothesis was supported in separate within group comparisons — paired *t*-tests — which showed that the improvement was significant for the control group but not for the experimental group. However, other statistical tests that compared the change in scores in the two groups did not show significant differences. One explanation for this might be the small sample size, relative to the observed effect size – the manipulation may not have been strong enough to produce conclusive differences between treatments.

Future research should ideally attempt to study this phenomenon with larger populations and in different sporting contexts. This would help to determine whether the effect of self-efficacy upon performance is replicable in other sport and performance tasks. The implications of finding that self-directed thoughts can impact performance negatively, even if those thoughts are not explicitly limited to performance expectancies or consciousness of one’s movements, but at assessments of self-efficacy and feedback that are generally considered helpful interventions, would be substantially impactful in the application of sports psychology to coaching protocol.

## Data availability statement

The raw data supporting the conclusions of this article will be made available by the authors, without undue reservation.

## Ethics statement

The studies involving humans were approved by Institutional Review Board, Ashoka University, Haryana, India. The studies were conducted in accordance with the local legislation and institutional requirements. Written informed consent for participation in this study was provided by the participants’ legal guardians/next of kin.

## Author contributions

All authors listed have made a substantial, direct, and intellectual contribution to the work and approved it for publication.

## Conflict of interest

The authors declare that the research was conducted in the absence of any commercial or financial relationships that could be construed as a potential conflict of interest.

## Publisher’s note

All claims expressed in this article are solely those of the authors and do not necessarily represent those of their affiliated organizations, or those of the publisher, the editors and the reviewers. Any product that may be evaluated in this article, or claim that may be made by its manufacturer, is not guaranteed or endorsed by the publisher.
