# Supplementary material for: Self-efficacy assessment hinders improvement on a deliberate cricket bowling practice task
Source: Front Psychol. 2023 Sep 19;14:1214767. doi: 10.3389/fpsyg.2023.1214767 (PMC10546038; doi:10.3389/fpsyg.2023.1214767)
Supplement: Supplementary file 1 [file Data_Sheet_1.PDF]

Appendix A

Questionnaire (A).

i. Name ______

ii. Age ______

iii. Experience of playing the sport

   i. How many years of formal training have you received in cricket?

      i. Less than 1
      ii. Between 1 and 3
      iii. Between 3 and 5
      iv. More than 5

   ii. How many hours in a week do you receive formal cricket training? (Write in relation to the last 3 months)

      i. Less than 2 hours
      ii. 2 to 4 hours
      iii. 4 to 6 hours
      iv. 6 to 8 hours
      v. More than 8 hours

   iii. How many hours do you spend practicing bowling in a week? (Write in relation to the last 3 months)

      i. Less than 2 hours
      ii. 2 to 4 hours
      iii. 4 to 6 hours
      iv. 6 to 8 hours
      v. More than 8 hours

Questionnaire (B).

i. Please rate your degree of confidence by recording a number from 0 to 100 using the scale below.

0	10	20	30	40	50	60	70	80	90	100

not at all moderately confident very confident

a. How confident are you of your ability of your ability as a bowler?

Confidence (0 - 100) _______

b. How confident are you of your ability of bowling 50% balls in the given target zone?

Confidence (0 - 100) _______

Questionnaire (C).

Q. Who is your favourite bowler? ________

Please rate their ability by recording a number from 0 to 100 using the scale below.

0	10	20	30	40	50	60	70	80	90	100

not at all moderately good very good

Q. How would you rate the ability of your favourite bowler?

Ability (0 - 100) _______

Appendix B

Table B1
ANOVA for the difference in bowling scores pooled across pre-test and post-test bowling, relative to participant gender.
ANOVA – Gender Differences in Overall Scores

**Cases**	**Sum of Squares**	**df**	**Mean Square**	**F**	**p**
Gender	23.246	1	23.246	3.144	0.084
Residual	295.730	40	7.393		

Note. Type III Sum of Squares

Table B2
ANOVA for the difference in task self-efficacy scores pooled for both experimental and control groups measured after the post-test, relative to participant gender.
ANOVA – Gender Differences in Task Self-Efficacy

**Cases**	**Sum of Squares**	**df**	**Mean Square**	**F**	**p**
Gender	1156.371	1	1156.371	1.660	0.205
Residual	27869.730	40	696.743		

Note. Type III Sum of Squares

Table B3

Repeated Measures ANOVA. Within Subjects Effects.

**Cases**	**Sum of Squares**	**df**	**Mean Square**	**F**	**p**
Test Time	12.964	1	12.964	7.131	0.011
Test Time*Condition	2.012	1	2.012	1.110	0.298
Residual	72.524	40	1.813		

Note. Type III Sum of Squares

Table B4
Repeated Measures ANOVA. Between Subjects Effects.

**Cases**	**Sum of Squares**	**df**	**Mean Square**	**F**	**p**
Condition	0.964	1	0.964	0.243	0.625
Residual	158.524	40	3.963		

Note. Type III Sum of Squares

Table B5. Correlation Matrix (Experimental Group)

Variable		Pre-test (E)	Post-test (E)
Pre-test (E)	Pearson's r		
	p-value		
Post-test (E)	Pearson's r	**0.490***	
	p-value	0.024	
Task Self-efficacy (1)	Pearson's r	**0.556****	0.119
	p-value	0.009	0.607
General Self-Efficacy (1)	Pearson's r	**0.582****	0.076
	p-value	0.006	0.745
General Self-Efficacy (2)	Pearson's r	0.563	0.180
	p-value	0.008	0.435
Task Self-Efficacy (2)	Pearson's r	**0.450***	0.237
	p-value	0.041	0.300

Table B6. Correlation Matrix (Control Group)

Variable		Pre-test (C)	Post-test (C)
Pre-test (C)	Pearson's r		
	p-value		

Post-test (C)	Pearson's r	0.194	
	p-value	0.400	
General Self-Efficacy	Pearson's r	0.362	0.207
	p-value	0.107	0.369
Task Self-Efficacy	Pearson's r	**0.531***	0.200
	p-value	0.013	0.385

Table B7. Descriptive statistics for bowling scores pooled across pre-test and post-test bowling, relative to participant gender.

	**Overall Score**	
	Female	Male
Valid	5	37
Missing	0	0
Mean	9.00	6.70
Std. Deviation	2.12	2.78
Shapiro-Wilk	0.910	0.954
P-value of Shapiro Wilk	0.468	0.126
Minimum	6	2
Maximum	11	14

Figure B1

Self-Efficacy Correlations

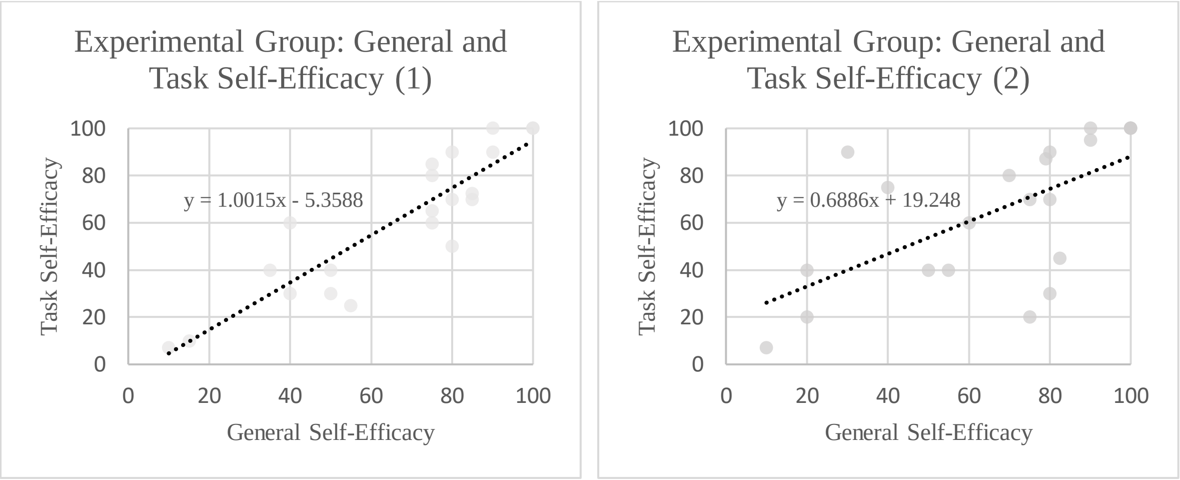

(A) (B)

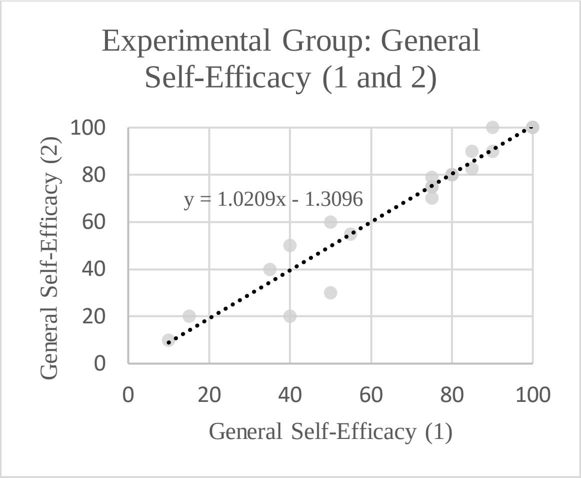

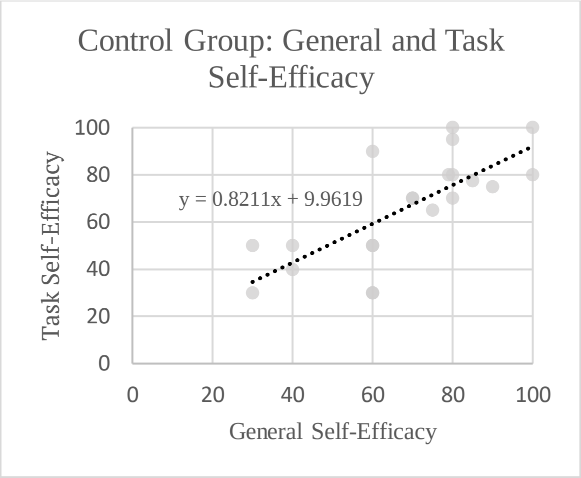

(C) (D)

Note. (A) The correlation between general efficacy and task-specific efficacy, both measured after the pre-test set of 12 trials (r = 0.90, p < 0.001) and (B) after the post-test set of 12 trials (r = 0.63, p = 0.002), in the experimental group. (C) The correlation between general self-efficacy measured after the pre-test (1) versus measured after post-test trials (2) for the experimental group (r = 0.96, p < .001). (D) The correlation between general and task specific efficacy in the control group (r = 0.74, p < 0.001).

Figure B2

Pre-test Scores and Self-Efficacy Correlations

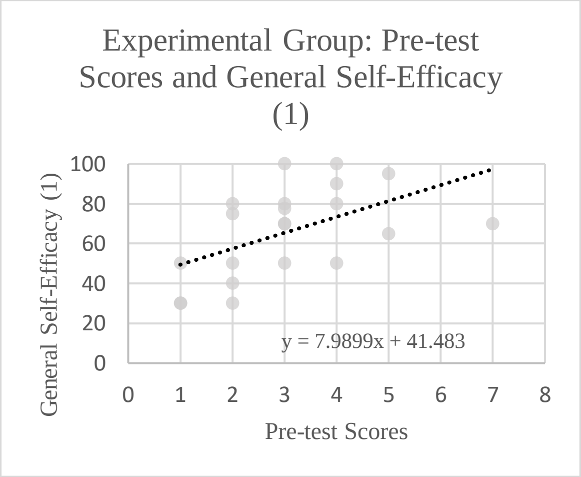

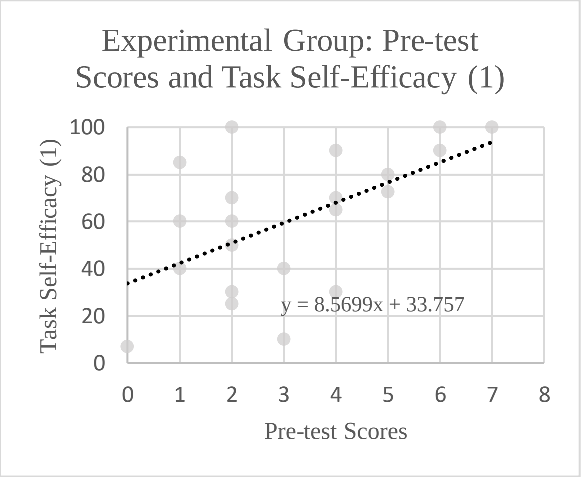

(A) (B)

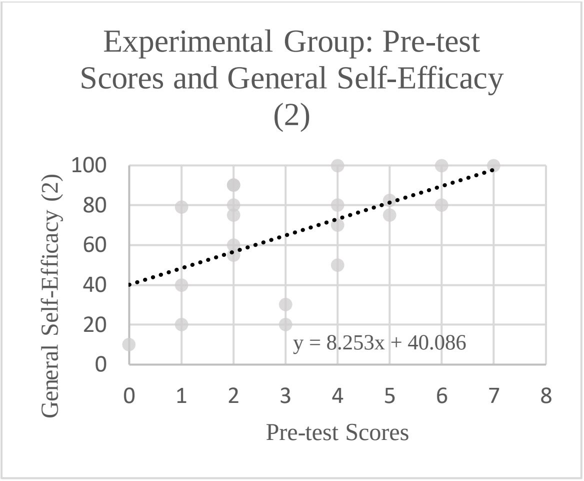

(C)

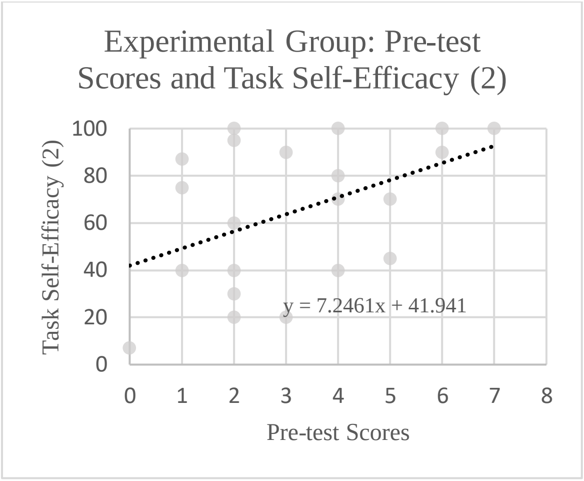

(D)

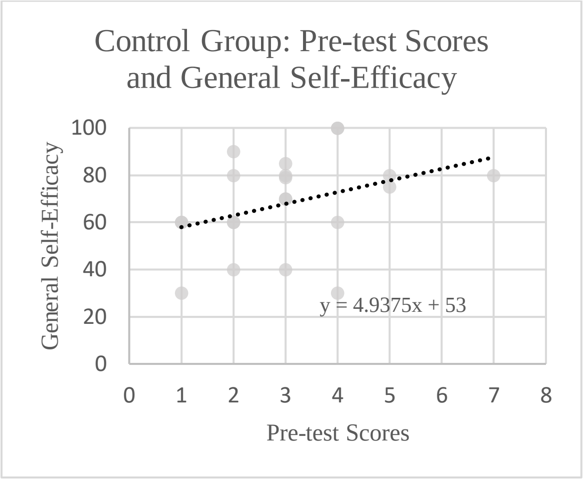

(E)

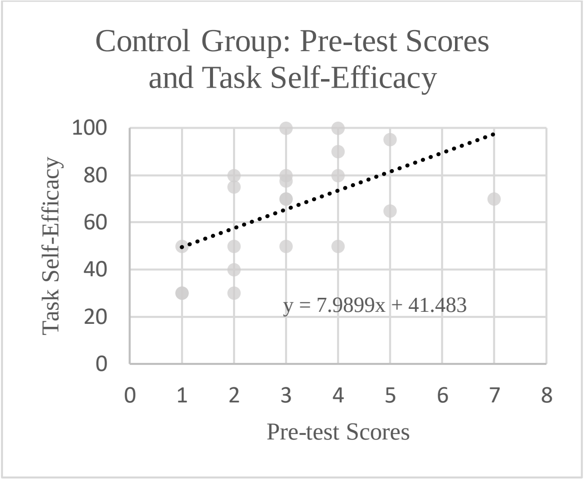

(F)

Note. Figure 5. The correlation between pre-test bowling scores, with (A) general efficacy (r = 0.58, p = 0.006) and (B) task-specific efficacy (r = 0.56, p = 0.009), both measured after the pre-test set of 12 trials and (C) general efficacy (r = 0.56, p = 0.008) and (D) task-specific efficacy (r = 0.450, p = 0.041), both measured after after the post-test set of 12 trials (r = 0.63, p = 0.002), in the experimental group; with (E) general self-efficacy (r = 0.36, p = 0.11) and (F) task-specific efficacy (r = 0.53, p = 0.013) in the control group.

Appendix C: Instructions to bowlers

"We want to study how a task can be learned. You will bowl four overs in total. After one over, you may take a break for thirty seconds. After two overs you will get a break for five minutes and will be asked to answer some questions. Then, you will bowl one warm up delivery. Finally, you will be asked to bowl the third and fourth over with an optional thirty second break in between. Your task is to pitch the ball in the area demarcated by the cones. Every time you pitch the ball in the area demarcated by the cones, it will be considered a successful trial. If you bowl a front foot no ball or bend your arm while bowling, the trial will not be counted. Do you have any questions?"
